# Genetic Diversity of Polymorphic Vaccine Candidate Antigens (Apical Membrane Antigen-1, Merozoite Surface Protein-3, and Erythrocyte Binding Antigen-175) in *Plasmodium falciparum* Isolates from Western and Central Africa

**DOI:** 10.4269/ajtmh.2011.10-0365

**Published:** 2011-02-04

**Authors:** Issiaka Soulama, Jude D. Bigoga, Magatte Ndiaye, Edith C. Bougouma, Josephine Quagraine, Prisca N. Casimiro, Timothy T. Stedman, Sodiomon B. Sirima

**Affiliations:** Centre National de Recherche et de Formation sur le Paludisme, Ouagadougou, Burkina Faso; Department of Biochemistry and the Biotechnology Center, University of Yaounde I, Yaounde, Cameroon; Service de Parasitologie–Mycologie, Université Cheikh Anta Diop, Dakar, Senegal; Department of Parasitology, Noguchi Memorial Institute for Medical Research, University of Ghana, Legon, Ghana; Centre d'Etudes des Ressources Végétales, Unité de Pharmacologie Brazzaville, Republic of Congo; Malaria Research and Reference Reagent Resource Center, American Type Culture Collection, Manassas, Virginia; Groupe d'Action et de Recherche en Santé, Belgium

## Abstract

The malaria vaccine candidate antigens erythrocyte binding antigen 175 (EBA-175), merozoite surface protein 3 (MSP-3), and apical membrane antigen (AMA-1) from *Plasmodium falciparum* isolates from countries in central and west Africa were assessed for allelic diversity. Samples were collected on filter paper from 600 *P. falciparum*-infected symptomatic patients in Cameroon, Republic of Congo, Burkina Faso, Ghana, and Senegal and screened for class-specific amplification fragments. Genetic diversity, assessed by mean heterozygosity, was comparable among countries. We detected a clinical increase in *eba 175 F-*allele frequency from west to east across the study region. No statistical difference in *msp-3* allele distribution between countries was observed. The *ama-1* 3D7 alleles were present at a lower frequency in central Africa than in West Africa. We also detected little to no genetic differentiation among sampling locations. This finding indicates that, at least at the level of resolution offered by restriction fragment length polymorphism analysis, these antigens showed remarkable genetic homogeneity throughout the region sampled, perhaps caused by balancing selection to maintain a diverse array of antigen haplotyes.

## Introduction

*Plasmodium falciparum* malaria is a major public health challenge and a serious impediment to socioeconomic development, particularly in sub-Saharan Africa, where 85% of the global burden occurs. An estimated 0.9 million deaths caused by malaria occur each year, and 89% of deaths occurr in children less than five years of age and in pregnant women living in malaria-endemic areas.^1^ Efforts expended to curb the burden with available antimalarial drugs, insecticides, and bed nets have met with some success. To be effective, a multifaceted malaria eradication program should include vaccination and effective control of mosquito vectors.^2^ The continued spread and persistence of *P. falciparum* malaria is caused in part by the development of anti-malarial drug resistance, primarily against those drugs targeting the parasite asexual erythrocytic stages. This stage is also responsible for pathologic and clinical manifestations of the disease.^3^ Thus, the need to develop alternative approaches to target this life stage with an effective vaccine is urgent.

The extensive genetic diversity of the malaria parasite constitutes major drawbacks to the development of a successful malaria vaccine.^4^,^5^ Such extensive antigenic polymorphism greatly enhances the parasites ability to evade immune recognition, making it difficult to elicit adequate responses against the full range of variants circulating in the parasite population.^6^ Fluctuations in genetic diversity across transmission seasons can further complicate control measures.^7^–^9^ Previous studies using microsatellite loci have shown significant geographic variation in genetic diversity between and within continents.^10^–^12^ An in-depth understanding of the distribution and dynamics of vaccine candidate antigen diversity in natural parasite populations is vital for designing a successful and broadly deployable malaria vaccine, as well as providing useful clues for interpretation of responses to the vaccine. Several *P. falciparum* stage-specific antigens have been characterized as vaccine candidates through molecular epidemiology. Characterization of the distribution of malaria parasite antigen genotypes circulating in a wide geographic area may provide important genetic information helpful for malaria vaccines design. We have analyzed the genetic diversity of three antigens with promise as vaccine candidates: erythrocyte binding antigen 175 (EBA-175), apical membrane antigen (AMA-1), and merozoite surface protein 3 (MSP-3).

*Plasmodium falciparum* EBA-175 antigen plays a central role in erythrocyte invasion by sialic acid–dependent binding to glycophorin A. It is well known that antibodies against EBA-175 inhibit binding to glycophorin A and thus prevent merozoite invasion of erythrocytes.^3^,^4^ The gene encoding EBA-175 is located on chromosome 7 and is comprised of four exons and seven regions termed I–VII that include three cysteine-rich regions (F1, F2, and C).^13^–^15^ Exon 1, located in region II, has four regions that encode a repeated sequence of two Duffy binding-like domains (F1 and F2). The two domains are separated by highly divergent dimorphic region III, through the insertion of either a 342-basepair segment in FCR-3 strains (F-loop) or a 423-basepair segment in CAMP strains (C-loop). The relevance of the F/C segments is not well known, but the proportions have been shown to vary among natural parasite populations in Africa.^16^,^17^ Previous studies analyzing the influence of this dimorphism on clinical status and outcomes in children from Gabon^15^,^17^ showed low prevalence of mixed C-/F-infection in asymptomatic children than in symptomatic persons.

Apical membrane antigen is an 83-kD type I integral membrane protein with a 55-amino acid cytoplasmic segment and a 550-amino acid extracellular region that can be divided into three domains on the basis of intra-domain disulfide bonds. Although its function is still not well characterized, it is expressed in the late schizont stage of the parasite and is required for merozoite invasion of erythrocytes and sporozoite invasion of hepatocytes. Antibodies against AMA-1 have been shown to block parasite invasion of human erythrocytes.^18^

*Plasmodium flaciparum ama-1* is highly polymorphic,^19^,^20^ and the domain I has been shown to contain most of the polymorphism.^19^,^21^ Based on restriction fragment length polymorphism (RFLP) analysis, domain I can be subdivided into four groups termed I, II, III, and IV.^20^ Protective responses induced by this antigen are strain specific, suggesting that these polymorphisms have arisen by diversifying selection.^22^

*Plasmodium falciparum* MSP-3 is encoded by a single locus on parasite chromosome 10, and is an important target for protective immunity because antibodies against it could prevent erythrocyte invasion by merozoites.^23^ Merozoite surface protein 3 is a member of a heteromeric protein complex associated with the merozoites outer membrane involved in interactions with the acidic basic repeat antigen, or ABRA (also known as MSP9).^23^–^25^ Structurally, the MSP-3 protein is composed of three blocks of tandemly alanine-repeated heptads and separated by short stretches of non-repetitive sequence unrelated to the heptad-repeat. Previous analysis of the *msp*-3 gene from *P. falciparum* isolates showed that polymorphism in the gene is predominantly confined to sequence in the N-terminal extremity within and flanking the heptad-repeat previously identified as a site of antigenic diversity among MSP-3 polypeptides.^24^ The C-terminal domain (corresponding to aa 196–379 in the K1 allele sequence) is highly conserved among various parasite isolates. 24–26 There are several sequences differences among *msp-*3 alleles, but the sequence polymorphism define 2 major allele classes (K1 like and 3D7 like), which show only limited recombination.^27^

The purpose of this study was to determine the distribution of major allele classes for three *P. falciparum* candidate vaccine antigens (AMA-1, EBA-175, and MSP-3) across varied eco-epidemiologic zones in central and West Africa. Field isolates were obtained from symptomatic persons in five countries and were typed with respect to vaccine antigen candidate major allele classes as defined by RFLPs. Knowledge of the distribution of polymorphic malaria antigens across this broad geographic range may contribute to the rational development of a malaria vaccine.

## Material and Methods

### Study sites and study population.

**Figure 1.**
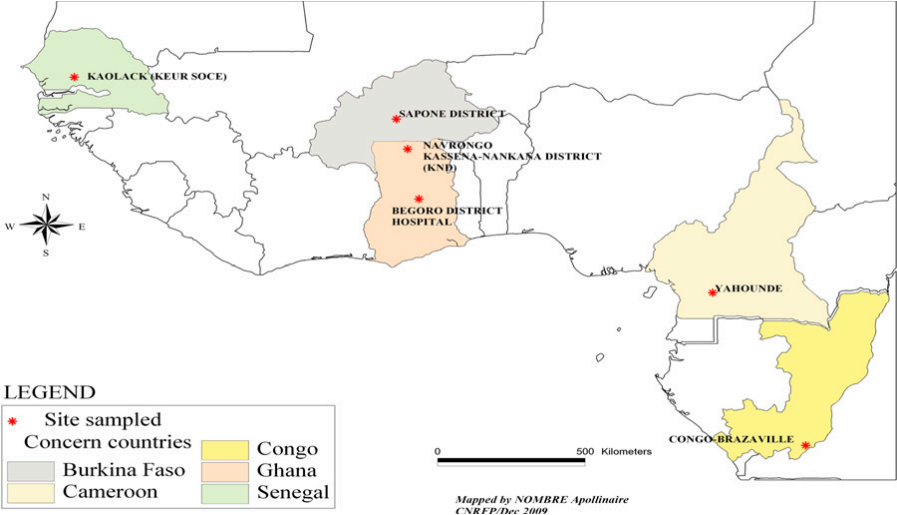
Map showing the sample collection sites and countries.

The study was conducted in different sites in five countries (Figure 1): Cameroon and Republic of Congo in central Africa; and Burkina Faso, Ghana, and Senegal in West Africa.

#### Burkina Faso.

Samples were collected from the Saponé Marché and Kounda communities health clinics located within an 8-km radius in the Saponé Health District situated approximately 50 km southwest of Ouagadougou, the capital of Burkina Faso. The Saponé Health District is located at 12°13′N, 1°48′W and has approximately 80,000 inhabitants, most (> 95%) from the Mossi ethnic group. The number of children less than five years of age in the health clinics area of the two communities was approximately 465. All malaria cases were freely managed by using artemisinin-based combination therapy (Coartem^®^; Roche, Basel, Switzerland) during a one-year longitudinal follow-up study. Malaria transmission is perennial and seasonal in the district, and there is a peak during the rainy season (May–October). The entomologic inoculation rate was estimated to be 200 infected/bites/person/year in the study area.^28^ The incidence of clinical malaria cases in children less than five years of age living in Saponé Health District was estimated to be 0.7 episodes/child-year (Sirima BS and others, unpublished data).

#### Cameroon.

The study was carried out in health centers in three localities (Nkolbisson, Etoug-egbe, and Mfou) in Yaounde. The three localities have equal malaria transmission intensities. Yaounde is located at 3°52′0″N, 11°31′0″E. The population is comprised of heterogeneous ethnic backgrounds from every region of the country. Malaria is the primary cause of morbidity in these localities. Maximal transmission (EIR > 200 infected bites/person/year) occurs during and immediately after the two rainy seasons (March–June and September–November).

#### Republic of Congo.

The study was conducted at the Tenrikyo Integrated Health Center, which is located within the Makélékélé Health District in Brazzaville, Republic of Congo. Brazzaville is located situated between 4°13′S and 4°18′S and 15°13′E and 15°18′E. The rainy season lasts eight months (October–May), and the dry season last four months (June–September).^29^ Malaria transmission is mediated mainly by *Anopheles gambiae*. Other vectors, such as *An. moucheti*, *An. funestus*, and *An. nili*, are also found.^30^

#### Ghana.

The study was conducted at the Navrongo War Memorial Hospital in the Kassena-Nankana District (northern Ghana) and the Begoro District Hospital in the Fanteakwa District (southern Ghana). The Kassena-Nankana District is located in the Upper East Region along the Ghana–Burkina Faso border. The district is hot and dry and is located in the Guinea Savannah Belt. Navrongo, the district capital, is located at 10°53′5″N, 1°5′25″W. Estimated malaria attack rates in the district are approximately 4.7 and 7.1 infections/person/year for the dry and wet seasons, respectively.^31^

The Fanteakwa District is located in the Eastern Region of Ghana. Begoro, which is the district capital of the Fanteakwa District, is located at 6°22′N, 0°23′W, approximately 150 km north of Accra, the capital of Ghana. Malaria is known as the most prevalent disease reported at the Begoro District Hospital.

#### Senegal.

The study took place in the rural community of Keur Socé located approximately 17 km south of Kaolack. Keur Socé is situated at 14°33′46.14″N, 15°35′30.53″W. Children less than five years of age represent approximately 20% of the population. Malaria transmission in this locality is seasonal. Entomologic inoculation rates vary between 9 and 12 infected bites/person/night during the high transmission season.

### Ethical considerations.

Ethical clearances and administrative authorizations were obtained by the competent Ethical Review Committees and issuing bodies for the respective studies with approval including consent to use samples for malaria related studies. These committees were the Health Research Ethics Review Committee in Burkina Faso, the National Ethic Committee in Cameroon, the Ministry of Health and the Ministry of Scientific Research in the Republic of Congo, the Institutional Review Board of the Noguchi Memorial Institute for Medical Research, University of Ghana, Legon, Ghana, and the Health Ethical Committee based at the Ministry of Health in Senegal. The purpose of the study and all anticipated risks and benefits were clearly explained to the participants. Written informed consent and assent were obtained from the participants or their guardians, and participation in the studies was strictly voluntary.

### Sample collection.

Sampling was conducted at clinics and hospitals. Samples collection criteria were *P. falciparum*-infected, symptomatic, voluntary children or infants with malaria living in the five study countries irrespective of parasite density. Symptomatic malaria includes uncomplicated malaria and severe malaria as per the World Health Organization malaria definition. To limit any bias related to data collection, samples were collected between during two consecutive years during 2007–2009. In Ghana, samples were obtained in 2007 and 2008 from children 6–60 months of age who were enrolled in a study to assess the therapeutic efficacy of anti-malaria drugs.^32^ In Burkina Faso, the study samples were obtained from children 6–59 months of age as part of a malaria morbidity and mortality longitudinal study carried out in 2007 and 2008. Samples from Senegal were obtained in 2007 and 2008 from children 6–59 months of age during an antimalarial drug clinical trial. In Cameroon, samples were obtained in 2009 from children less than 15 years of age involved in a study aimed at assessing the genetic diversity of polymorphic malaria candidate vaccine antigens. In Congo, samples were obtained from children less than 15 years of age during a sulfadoxine-pyrimethamine and chloroquine efficacy assessment in 2009.

Samples were obtained at clinics or hospitals from 659 *P. falciparum*-infected symptomatic children with malaria living in five countries in central and west Africa: 231 isolates from Saponé Health District (Burkina Faso), 105 isolates from Keur Socé (Senegal), 122 isolates from Yaounde (Cameroon), 100 isolates from the Republic of Congo, and 101 isolates from Begoro (southern Ghana) and Navrongo District Hospitals (northern Ghana).

Thick and thin blood smears were prepared for microscopic identification of *P. falciparum*. Whole blood from *P. falciparum*-positive persons was blotted on sterile, Whatman (Maidstone, United Kingdom) III filter paper, stored dry at room temperature and protected with a silica gel desiccant, and archived for later *P. falciparum* DNA isolation.

### Microscopic examination.

Giemsa-stained blood smears were microscopically examined to identify mono-infections with *P. falciparum*. Slides were examined by experienced microscopists in each study site. A person was considered positive if malaria parasites were detected in the blood smear and negative if parasites were not detected after examining 200 oil-immersion fields of a thick blood smear.

### Extraction of *P. falciparum* DNA.

*Plasmodium falciparum* DNA was isolated from archived blood spots by using two methods: Tris-EDTA (TE) with heat^33^ and the Qiagen (Valencia, CA) blood blot extraction method with QIAmp DNA blood mini kits. For the TE and heat method, approximately 5 mm of the blood spot per sample was cut with a sterile disposable blade and placed in 1.5 mL Eppendorf (Hamburg, Germany) tube. Sixty-five microliters of TE buffer (10 mM Tris, pH 8.0, 0.1 mM EDTA in distilled water) was added and the tube was incubated at 50°C for 15 minutes. The paper was gently pressed several times at the bottom of the tube with a new and sterile pipette tip for each sample. The tube was again incubated at 95°C for 20 minutes and centrifuged for 3 seconds to remove condensation from the lid. The DNA extracts were aliquoted into sterile tubes. Extraction by the Qiagen kit method was conducted according to the manufacturer's protocol. All DNA samples were stored at –20°C before genotyping with a polymerase chain reaction.

### PCR amplification.

For *eba-175*, nested PCR products were analyzed for amplification fragment length polymorphisms.^34^ For *msp-3*, semi-nested PCR was used to produce DNA fragments. The *ama-1* haplotypes were analyzed by using PCR-RFLP.^20^ Oligonucleotide primer sequences for the primary and secondary amplifications are listed in Supplemental Table 1.

The first-round PCR amplifications were performed in a final volume of 25 μL that contained 1 μM of each primer, 1 unit of Taq DNA polymerase (Invitrogen, Carlsbad, CA), 2.5 μL of 10× PCR buffer (Invitrogen), 2.0 mM MgCl_2_, 200 μM dNTP (Invitrogen), and 5 μL of DNA template extracted from blood. The second round amplification reactions were performed similarly to the first with 2.5 μL of the first PCR product as DNA template in 25 μL total reaction volume (*eba-175* and *msp-3*). Second-round reaction mixtures for *ama-1* were performed in a final volume of 50 μL that contained 5 μL of 10× PCR buffer (Invitrogen), 2 units of Taq DNA polymerase (Invitrogen), and 5 μL of DNA template. All reactions were performed using 96-wells plate on a classic 96-well thermocycler (Eppendorf).

Cycling conditions for the first and second PCR cycles of *eba-175* and *msp-3* were 94°C for 30 seconds, 54°C for 30 seconds, and 68°C for 2 minutes, followed by a final extension at 68°C for 5 minutes, for a total of 30 and 40 cycles respectively. For *ama-1*, the amplification cycles were 95°C for 2 minutes 30 seconds, 51°C for 30 seconds, and 68°C for 45 seconds, followed by a final extension at 68°C for 5 minutes for 30 cycles for the first nested PCR and 35 cycles for the second PCR.

Purified DNA from *P. falciparum* 3D7 (MRA-102G), HB3-B2 (MRA-149G), Dd2 (MRA-152G), 7G8 (MRA-153G), W2 (MRA-157G), K1 (MRA-159), V1/S (MRA-176), and FCR3 (MRA-731) strains was provided by the Malaria Research and Reference Reagent Resource Center, American Type Culture Collection (Manassas, VA) and used as positive controls during the amplification reactions.

The second amplification products from *eba-175* and *msp-3* were directly separated by electrophoresis on a 2.0% ethidium bromide agarose gel and visualized on a GEL LOGIC 220 UV-Transillumination Imaging System (Carestream, Rochester, NY). The second *ama-1* amplification product was digested by using three restriction enzymes (*Mse* I, *Ssp* I, and *BfCU* I), which generated digestion fragments specific for the D7, K1, and HB3/7G8 *ama-1* allele classes, respectively.

### Data interpretation.

Positive controls were used to scored and interpreted fragment sizes. The *msp-3* K1 allele was identified as a single approximately 500-basepair fragment, and the *msp-3* 3D7 allele was identified as a single approximately 400-basepair fragment. Any other *msp-3* fragments of different sizes were also reported. The CAMP allele was identified as a single approximately 714-basepair fragment, and the FCR3 allele was identified as a single approximately 795-basepair fragment. Mixed infections were defined as the simultaneous presence of K1 and 3D7 *msp-3* alleles or the F and C *eba-175* fragments in the same sample. The *ama-1* K1, 3D7, and HB3/7G8 alleles were identified as single fragments of 285, 400, and 335 basepairs, respectively, when subjected to digestion with *Mse* I, *Ssp* I, and *BfuC* I, respectively.

### Statistical analysis.

Allelic data were analyzed by using GenAlex 6,^35^ Arlequin v3.1,^36^ and Epi Info v6.04a (Centers for Disease Control and Prevention, Atlanta, GA). Data were analyzed by using chi-square or Fisher's exact tests; we used a statistical significance threshold of *P* < 0.05. Allele frequencies were estimated in GenAlEx (http://www.anu.edu.au/BoZo/GenAlEx/). Genetic diversity was estimated by determining the heterozygosity based on the three antigens combined, where heterozygosity (*h*) = 1 − ∑ *pi*^2^ ranged from 0 to 1, where *pi* is the frequency of the *i-^th^* allele. The *t*-test was used to compare mean heterozyosity among the different sites.

To compare allele frequencies along an east–west transect, data were pooled into three geographic groups: Cameroon and Republic of Congo (east), Burkina Faso, southern and northern Ghana (middle) and Senegal (west).

We used Nei's standard genetic distance, and the Mantel test to measure the genetic differentiation of genes across the different study sites. The option of the Mantel test we used enables a statistical relationship between the elements of any two distance matrices with matching entries. This typical application includes testing for isolation-by-distance for which one might compare Nei genetic distance matrix with the geographic distance matrix for the respective populations. Typically, it is reported as an XY graph with log-distance versus linearized Fest (in our case using binary data it is linear zed-Phi). We examined how genetic variation was partitioned among geographic units by using analysis of molecular variance.^37^

## Results

The main goal of this study was to analyze separately and in combination three polymorphic antigen genes across west and central Africa to identify differences in allele frequency and genetic diversity and to determine whether geographic distance contributes to genetic differentiation across our study region.

Of the 659 confirmed *P. falciparum* samples obtained from the five countries, 418 samples were successfully scored for *eba-175*, 535 for *msp-3*, and 318 for *ama-1*. Two hundred sixty-six samples were used in the combined datasets analyses. To minimize the confounding effects of missing data, inclusion in the combined dataset was limited to samples with missing data for no more than one marker. Failure to amplify domains from a fraction of the patient samples (241 for *eba-175*; 124 for *msp-3*, and 341 for *ama-1*) was attributed to variance in parasitemia, quality of the filter paper used, and extraction efficiency from the paper. All these PCR failures were excluded from further analysis.

A total of 97 mixed infections, or 15% of all infections sampled, were identified on the basis of multiple *eba*-175 or *msp*-3 alleles. This number is likely an underestimation because all markers were not successfully scored for all isolates. Most of the mixed infections (> 40%) were from rural villages in Burkina Faso. Mixed infections were included in all further analyses.

### Allele prevalences across the study area.

**Figure 2.**
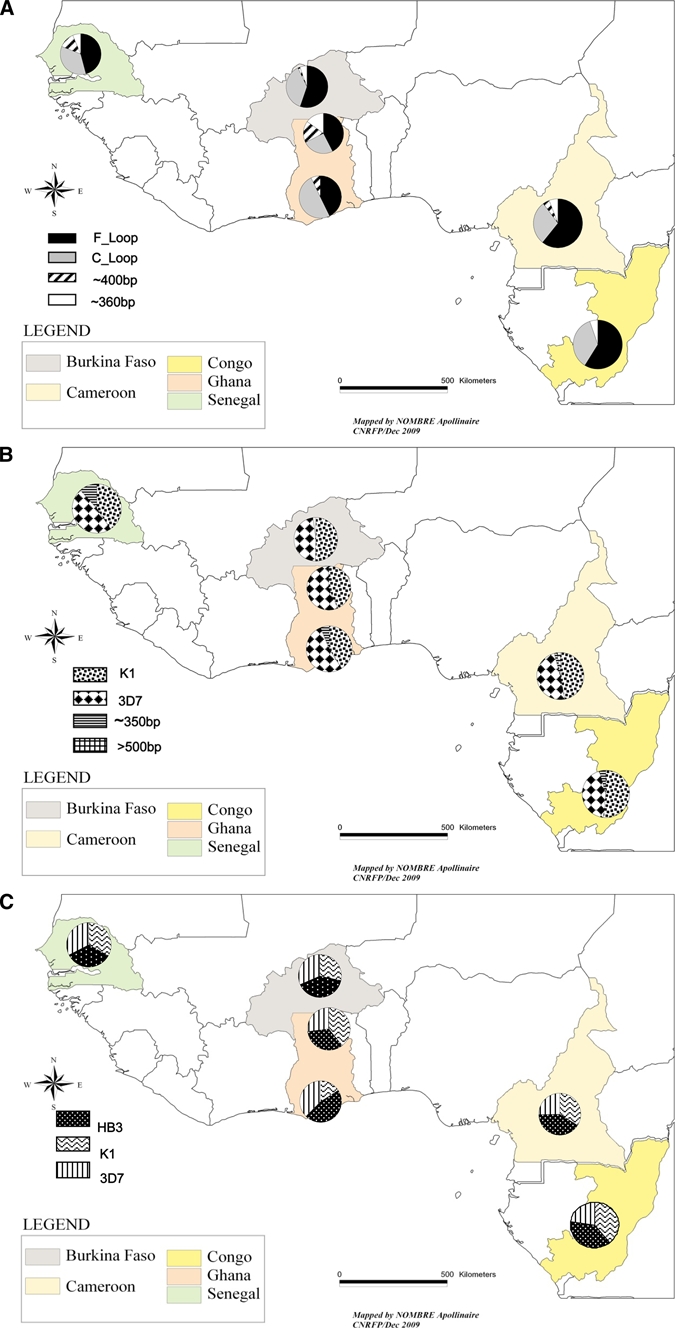
**A**, Distribution of erythrocyte binding antigen-175 alleles. **B**, Distribution of merozoite surface protein-3 alleles. **C**, Distribution of apical membrane antigen-1 alleles.

Allele prevalences were analyzed for each locus to assess variation across the study area. The F-loop allele accounted for > 50% of *eba-175* alleles in Burkina Faso, Republic of Congo, and Cameroon, where it was twice as common as the C-loop allele (Table 1). In general, the F-loop allele showed a clinal decrease in prevalence moving from east to west (Figure 2A). No such trends in allele prevalence were observed for the other two markers, and they appeared to be remarkably consistent across the study area (Figure 2B and C). Rare *eba*-175 alleles were observed in most but not all study sites. The *eba-175* 400 bp allele was more prevalent in Ghana and Senegal (> 10%) than in Burkina Faso, Cameroon, and Republic of Congo (< 2%) (Figure 2A). Despite the trend observed for the F-loop allele, there was no statistical difference in the *eba-175* allele prevalence among countries or between geographic regions, with the exception of the 400_bp_ rare allele, which was significantly more common in southern Ghana (Table 1 and Figure 2A).

The *msp-3* allele classes (K1 and 3D7 types) showed comparable prevalence in all countries (Table 1 and Figure 2B). In contrast, the rare 350_bp_ allele was observed in all countries but was approximately three times more common in Senegal (prevalence = 11%) (Table 1 and Figure 2B). The other rare *msp-*3 allele appeared to be restricted to Cameroon and accounted for only 2% of alleles from that country (Table 1 and Figure 2B). In addition, the comparison of *ama-1* alleles prevalence among geographic regions showed a significant difference (*P* = 0.03) in the *ama-1* 3D7 allele prevalence and showed a lower prevalence in the eastern portion of the study region (Republic of Congo and Cameroon) (Table 1 and Figure 2C).

### Antigen genetic diversity.

**Figure 3.**
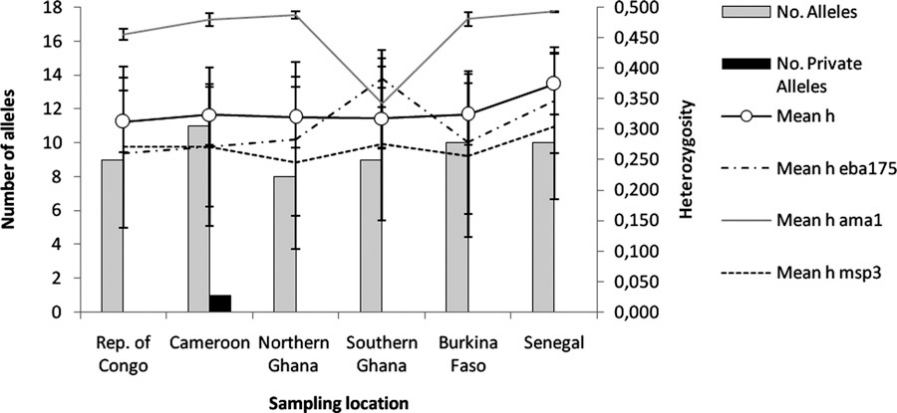
Mean heterozygosity and number of alleles of erythrocyte binding antigen-175 alleles, merozoite surface protein-3 alleles, and apical membrane antigen-1 alleles at each sampling location.

Mean heterozygosity (*h*) was calculated from allele frequencies to compare genetic diversity in the different study sites. For the combined dataset, the number of alleles ranged from 8 to 10. One *msp-3* allele, (*msp3* > 500_bp_) was exclusively present in Cameroon (Figure 3). The mean heterozygosity for the combined dataset did not differ greatly among the study sites (range = 0.313–0.375). In general, the heterozygosity in *ama-1* was higher than in the other two genes. In addition, individual gene heterozygosities did not differ among study sites, with the exception of southern Ghana (Figure 3), where there was a noticeable and significant decrease *ama-1* heterozygosity (*P* < 0.0001, by *t*-test) and a corresponding spike for *eba-175* (*P* < 0.001).

The overall heterozygosity was significantly higher in Senegal (*P* < 0.0001, by *t*-test) and for *ama-1*, *eba-175*, and *msp3* individually. However, because the southern Ghana sample size (5.6% combined [15 of 266]) was small (n = 15, combined marker dataset), our heterozygosity estimate for this site is probably not an accurate reflection of the true value.

### Genetic differentiation of antigen across the study area.

**Figure 4.**
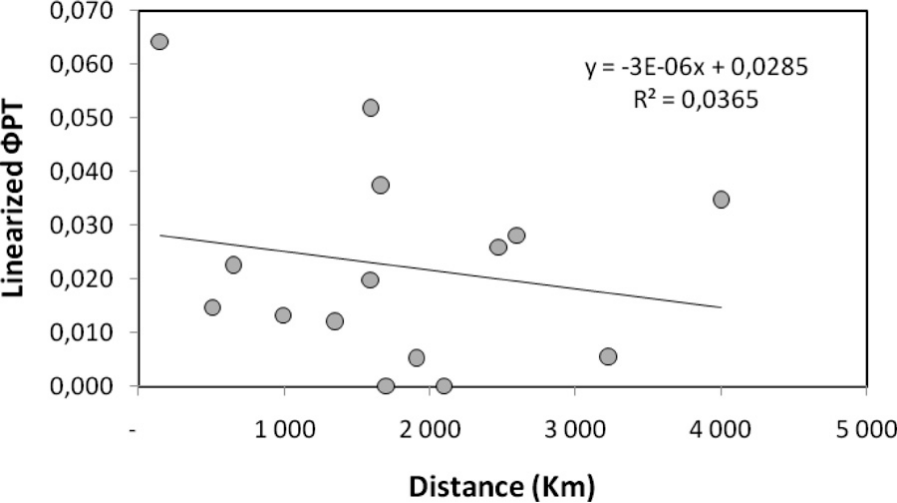
Isolation by distance (*Φ*_PT_) between populations. *Φ*_PT_ is a measure of population genetic differentiation for binary data and is analogous to *F*_st_.

Analysis of molecular variance enabled hierarchical partitioning of genetic variation within and among study sites and among pre-defined geographic regions (west: Senegal; middle: northern Ghana, southern Ghana, and Burkina Faso; east: Republic of Congo and Cameroon). Most genetic variation was captured within individual populations (97%), and there was little variance among a population within a region (3%) and no partitioning among geographic regions (Table 2). Nei's Standard Genetic Distance was used to assess genetic differentiation among study sites. In general, genetic distance values were low (approximately 0; Table 3). Genetic distance between southern Ghana and the others study sites was generally higher, although still approximately 0 (Table 3). The Mantel test was used to assess the influence of geographic distance on the genetic diversity of the antigens sampled. It showed a non-significant trend of greater genetic distance with decreasing geographic distance. Close examination of the results showed that a single data point, representing the comparison of northern Ghana with Burkina Faso, is primarily responsible for this trend because these two sites are genetically distinct but geographically close (approximately 135 km) (Figure 4). These analyses indicate that the major allele classes are homogeneously distribution across the study region.

## Discussion

*Plasmodium falciparum* is known to exhibit extensive genetic diversity, particularly among surface antigens that have been under selective immune pressure and historically considered as the main targets of subunit vaccines. Unfortunately, this extreme genetic diversity poses a major challenge for effective vaccine development because it could lead to vaccine-resistant malaria with non-vaccine type parasites growing in frequency within vaccinated populations.^5^ Therefore, to design a vaccine that is efficacious against any natural *P. falciparum* infection, multiple allelic forms of an antigen need to be incorporated in a vaccine formulation.^19^,^38^

The present study was designed to assess the distribution of major allele classes for three malaria vaccine candidates in a number of study sites across a geographic region spanning central and West Africa. Although collection protocols were not standardized across study sites, active case detection was used at all sites, and samples were collected within the same three-year time frame. In addition, all molecular methods and gel interpretations were standardized and conducted in the same laboratory at the same time. The markers used in this study did not have the resolution to measure the extensive genetic diversity known to exist for these surface antigens, but rather provides a pre-vaccine, baseline assessment of the distribution of major allelic classes. Our results address the feasibility of deploying a single vaccine throughout this large geographic region, and highlight the challenges posed by the considerable genetic diversity of these surface antigens.

Although the *eba-175* F-loop allele clearly predominates in the eastern portion of our study region (Cameroon and Republic of Congo), there is no one allele that is most common throughout the rest of the study area. The predominance observed in F-loop allele was similar to previous observations made in other parts of Africa.^15^,^17^,^39^ In Senegal, frequencies of the two alleles (C-loop and F-loop) were not statistically different, which is consistent with a previous report from central and southern Senegal.^40^

The *msp-3* allele classes (K1 and 3D7 type)^26^ result from many amino acid substitutions. These two major allelic classes showed a balancing frequency throughout the study region, and this was also reflected in the equivalent *msp-3* heterozygosity values observed across sites. We observed additional but less common, *msp-3* alleles (350 bp and 500 bp), which highlights the existence of significant alternative polymorphisms, as suggested by Polley and others*.*^41^ The comparable diversity among countries in the present study confirmed previous results showing that the major *msp-3* dimorphic allele classes were maintained intact at a low frequency of recombination.^41^

The isolates of *P. falciparum* in our study belonged to three of the four *ama-1* allele classes previously identified based on the hypervariable region. The fourth allelic class was not observed in our study and is presumed absent in sub-Saharan Africa. Polymorphism analysis of *ama-1* in the Amazon Basin in Peru identified three of the four *ama-1* allelic classes,^11^ and analyses in India identified only two classes.^42^ In the present study, the three alleles had roughly equivalent frequencies throughout the study region, with the exception of northern Ghana. Pooling data by region showed that the *ama-1* 3D7-like allele was less common in the eastern region, which may have been caused by a directional selection for humoral immune response.^19^,^40^ In general, *ama-1* diversity was high and comparable across the study sites even where there were regional differences in the prevalence of the different allelic classes and within-class variability, which may pose a problem for a vaccine based on this antigen. Although a remarkable conservation of the AMA-l molecule has been previously reported,^20^,^43^ it is important to understand the extent to which variation in *ama-1*, which we were not able to detect by using RFLPs in the present study, may compromise vaccine development. It is well known that the total repertoire of circulating *ama-1* alleles is probably large.^20^

Most of the observed genetic variance was not related to regional difference but attributed to variation within study sites. In addition, geographic distance did not contribute to antigen genetic distance. It is highly unlike that unrestricted gene flow across such a large geographic region is responsible for these patterns. Our data showed the presence of the main allele classes of *eba-175*, *msp-3* and *ama-1* at high and comparable frequencies throughout the study region. The lack of genetic differentiation or isolation-by-distance is probably caused by balancing selection. It is likely that immune selection for maximal diversity has overwhelmed any geographic partitioning we might otherwise have observed by using neutral markers.

Each of the target loci is currently in vaccine trials intended for populations in sub-Saharan Africa where the number of malaria cases identified in the past 10 years shows little evidence of decreasing.^1^ The genetic diversity and relatively balanced distribution of allele classes provides evidence that an effective vaccine should necessarily account for different allelic classes in combination, although this could be challenging with regards to product development.^44^ If vaccines based on universally conserved subunit targets were to be used, field monitoring of known and novel allelic polymorphisms associated with the target before and after vaccination will provide important information on vaccine epidemiology and immune selection.

## Supplementary Material

Supplemental Table

[Supplemental Table]

## Figures and Tables

**Table 1:** Erythrocyte binding antigen-175, merozoite surface protein-3, and apical membrane antigen allele prevalence for countries and regions in Africa

Allele*	East	Middle	West	Southern Ghana, no. (%)	Burkina Faso, no. (%)	Senegal, no. (%)	*P* (comparison of all sites)	West region, no. (%)	Middle region, no. (%)	East region, no. (%)	*P*†
	Republic of Congo, no. (%)	Cameroon, no. (%)	Northern Ghana, no. (%)								
eba175_F_loop	33 (58.9)	42 (60.9)	12 (46.2)	17 (50)	91 (55.2)	51(45.9)	0.5	51 (45.9)	120 (51.5)	75 (60.0)	0.09
eba175_C_loop	20 (35.7)	20 (29.0)	14 (53.8)	10 (29.4)	62 (37.6)	39 (35.1)	0.2	39 (35.1)	86 (36.9)	40 (32.0)	0.7
eba175–400bp	0 (0)	3 (4.3)	2 (0.08)	7 (20.6)	3 (1.8)	14 (12.6)	0.00001‡	14 (12.6)	12 (5.2)	3 (2.4)	0.003‡
eba175–360bp	3 (5.4)	4 (5.8)	0 (0)	6 (17.6)	9 (5.5)	7 (6.3)	0.1	7 (6.3)	15 (6.4)	7 (5.6)	*0.9*
msp3_K1	46 (51.7)	44 (46.8)	16 (44.4)	20 (44.4)	111 (50.2)	45 (40.5)	0.2	45 (40.5)	147 (47.9)	90 (49.2)	*0.4*
msp3_3D7	39 (43.8)	46 (48.9)	21 (58.3)	26 (57.8)	105 (47.5)	53 (47.8)	0.7	53 (47.8)	152 (49.5)	85 (46.4)	*0.8*
msp3–350bp	4 (4.5)	2 (2.1)	0 (0)	3 (0.06)	5 (2.3)	13 (11.7)	0.002‡	13 (11.7)	8 (2.6)	6 (3.3)	0.0002‡
msp3–500bp	0 (0)	2 (2.1)	0 (0)	0 (0)	0 (0)	0 (0)	–	0 (0)	0 (0)	2 (1.1)	–
ama1_K1	42 (40.8)	44 (37.6)	14 (63.6)	14 (38.9)	33 (27.3)	30 (37.0)	0.003‡	30 (37.0)		86 (39.1)	0.1
ama1_HB3	39 (37.9)	45 (38.5)	9 (52.9)	20 (74.1)	47 (38.8)	25 (30.9)	0.06	25 (30.9)	76 (36.7)	84 (38.2)	0.5
ama1_3D7	22 (21.4)	28 (23.9)	8 (36.4)	21 (63.4)	41 (33.9)	26 (32.1)	0.2	26 (32.1)	70 (33.8)	50 (22.7)	0.03‡

* eba175 = erythrocyte binding antigen 175; bp = basepair; msp3 = merozoite surface protein 3; ama1 = apical membrane antigen-1.

† *P* value for comparison of allele frequency distributions among the three regions: East versus Middle versus West.

‡ Significant *P* values.

**Table 2:** Hierarchical analysis of genetic variance among regions in Africa (Senegal, Burkina Faso, and Ghana, and Cameroon and Republic of Congo) and populations within regions

Variance component, variance %, total *P*
Among regions: 0, 0%, 0.880
Among populations within regions: 0.06, 3%, 0.030
Within populations: 2.203, 97%, 0.020

**Table 3:** Pairwise population matrix of Nei's genetic distance among sampling locations in Africa

Republic of Congo	Cameroon	Binary combined data				
		Northern Ghana	Southern Ghana	Burkina Faso	Senegal	Country
0.000						Republic of Congo
0.019	0.000					Cameroon
0.018	0.029	0.000				Northern Ghana
0.054	0.069	0.057	0.000			Southern Ghana
0.030	0.006	0.037	0.075	0.000		Burkina Faso
0.034	0.013	0.028	0.064	0.016	0.000	Senegal
