# Supplementary material for: Genetic Diversity of Polymorphic Vaccine Candidate Antigens (Apical Membrane Antigen-1, Merozoite Surface Protein-3, and Erythrocyte Binding Antigen-175) in Plasmodium falciparum Isolates from Western and Central Africa
Source: Am J Trop Med Hyg. 2011 Feb 4;84(2):276–84. doi: 10.4269/ajtmh.2011.10-0365 (PMC3029182; doi:10.4269/ajtmh.2011.10-0365)
Supplement: Supplemental Table [file SD3.pdf]

The following are supplemental materials and will be published online only

Supplemental Table 1

Primers and sequences used in the study, Africa*

Primer name	Sequence length 5′→3′
For the first amplification reactions, the following primers were used	
eba1 (forward)	CAAGAAGCAGTTCCTGAGGAA
eba-2 (reverse)	TCTCAACATTCATATTAACAATTC
msp-3 (159F)	ATGTTGCTAGTAAAGAAATTG
msp-3 (745R)	CATAACTAGAAGCTTCTTTTGC
ama-1 (VM785/3)	CCGGATCCCCTTTGAGTTTACATATATG
ama-1 (VM990)	AAATTCTTTCTAGGGCAAAC
For the second amplification reaction, the following primers were used	
eba-3 (forward)	GAGGAAAACACTGAAATAGCACAC
eba-4 (reverse)	CAATTCCTCC-AGACTGTTGAACAT
msp-3 (188F)	ATAATCTTAACTTAAGAAATGC
msp-3 (745 R)	CATAACTAGAAGCTTCTTTTGC
ama-1 (VM815)	GGAACTCAATATAGACTTCC
ama-1 (VM990)	AAATTCTTTCTAGGGCAAAC

* *eba* = erythrocyte binding antigen; *msp* = merozoite surface protein; *ama* = apical membrane antigen.
