# Supplementary material for: Genetic Diversity of Polymorphic Vaccine Candidate Antigens (Apical Membrane Antigen-1, Merozoite Surface Protein-3, and Erythrocyte Binding Antigen-175) in Plasmodium falciparum Isolates from Western and Central Africa
Source: Am J Trop Med Hyg. 2011 Feb 4;84(2):276–84. doi: 10.4269/ajtmh.2011.10-0365 (PMC3029182; doi:10.4269/ajtmh.2011.10-0365)
Supplement: [Supplemental Table] [file supp_84_2_276__index.html]

 Genetic Diversity of Polymorphic Vaccine Candidate Antigens (Apical Membrane Antigen-1, Merozoite Surface Protein-3, and Erythrocyte Binding Antigen-175) in Plasmodium falciparum Isolates from Western and Central Africa -- Soulama et al. 84 (2): 276 Data Supplement - Supplemental Table -- American Journal of Tropical Medicine and Hygiene **Genetic Diversity of Polymorphic Vaccine Candidate Antigens (Apical Membrane Antigen-1, Merozoite Surface Protein-3, and Erythrocyte Binding Antigen-175) in *Plasmodium falciparum* Isolates from Western and Central Africa**  
 Am J Trop Med Hyg Soulama et al. 84: 276

## Supplemental Table

**Files in this Data Supplement:**

- Supplemental Table
